# DNA-aptamer gating membranes†
†Electronic supplementary information (ESI) available. See DOI: 10.1039/c4cc09660f



**DOI:** 10.1039/c4cc09660f

**Published:** 2015-01-30

**Authors:** Thomas Schäfer, Veli Cengiz Özalp

**Affiliations:** a POLYMAT , University of the Basque Country , Av. Tolosa 72 , 20018 Donostia-San Sebastián , Spain . Email: thomas.schafer@ehu.es ; Fax: +34 943 01 7065 ; Tel: +34 943 01 8266/8399; b Ikerbasque , Basque Foundation for Science , Bilbao , Spain; c School of Medicine , Istanbul Kemerburgaz University , 34217 Istanbul , Turkey

## Abstract

This report describes a membrane barrier whose permeability is modulated through the recognition of a small-molecule target, adenosine triphosphate (ATP), by a DNA-aptamer. The gating function of the DNA-aptamer in the stimulus-responsive membrane was shown to be specific, concentration dependent, and reversible.

## Abstract 2

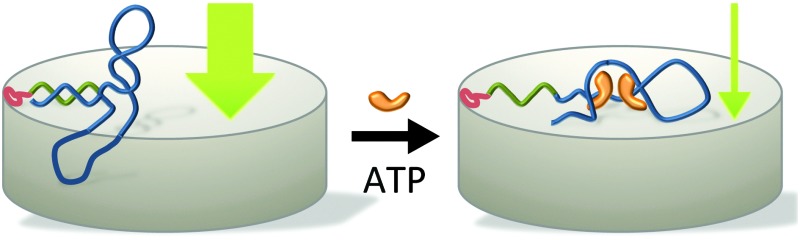
This report describes a membrane barrier whose permeability is modulated through the recognition of a small-molecule target, adenosine triphosphate (ATP), by a DNA-aptamer.

Approaches to creating stimulus-responsive membranes have been explored for decades for liquid separations or controlled release applications yielding materials whose permeability varies, triggered by a change of pH, temperature or ionic strength of the adjacent liquid, or the exposure to light, an electrical or a magnetic field.^1^ It has remained a challenge, however, to mimic the specific and locally acting molecular recognition mechanism which Nature employs for reversibly triggering a conformational change of a membrane receptor molecule in order to bring about a variation of the permeability of a cell membrane.^2^ Bioconjugated membranes incorporating enzymes or antibodies^3^ have marked an important milestone toward this goal; however, their complexity is far higher than that of, for example, oligonucleic acids or DNA-aptamers which in turn offer a potentially wider range of possible targets.^4^

DNA-aptamers have been successfully employed as building blocks in controlled release platforms where a molecular recognition event results in an irreversible opening of a mesoporous support structure, followed by the release of the cargo molecules.^5^ The concept was then further developed to yield DNA-aptamer based nanogates that serve as both recognition elements and reversible actuators in mesoporous silica nanoparticles, providing a reusable, concentration-dependent controlled release platform.^6^ The ultimate goal of such systems it to bring this proof-of-concept to function in membrane barriers, mimicking in this way the function of biological membranes. The challenge is hereby to transform the conformational change of the DNA-aptamer into an efficient way of modulating the permeation of tracer molecules across a membrane pore. We here report on such a self-assembled stimuli-responsive membrane barrier whose permeability can be modulated through a molecular recognition event and subsequent conformational change of an adequate DNA-aptamer. With the stimulus being a target molecule as small as adenosine 5′-triphosphate (ATP), the function of our stimulus-responsive membrane relies on the conformational change that aptamers undergo upon the specific recognition of this target molecule, rather than acting upon a bulk stimulus.

The modular architecture of the membrane comprised as a mesoporous base-material an anodized aluminum oxide (AAO) membrane of a nominal pore size of 20 nm and a narrow pore-size distribution (ESI,† Fig. E1). AAO membranes possess a high pore-density and therefore allowed assembling the responsive membrane in a more reproducible and controlled manner than when using polymeric membranes because the latter generally possess a wider pore-size distribution and less defined pore geometry. Track-etched membranes as support structures were also discarded given their very low pore density that would result in an unfavourable ratio of actuating to overall immobilized DNA-aptamer. We functionalized the AAO membrane surface was with amino groups through an ethoxysilane monolayer deposition^7^ on top of which we deposited avidin as a support layer through a biotin-linker (Fig. 1). This avidin layer served the purpose of providing a most favourable support layer for the subsequent immobilization of the biotinylated ATP-binding aptamer. Owing to the strong interaction between avidin and biotin, we assured in this way a highest possible immobilization density of the ATP-binding aptamer as the receptor molecule of the responsive membrane (for details see ESI,† Table S1). The ATP aptamer sequence used in our study (CACCTGGGGGAGTATTGCGGAGGAAGGTTCCAGGTG) was based on that reported by Huizenga and Szostak^8^ and shown previously to bind specifically two ATP molecules.^9^ It has been successfully employed in mesoporous particle-based controlled release systems.^6,10^ For the purpose of amplifying its conformational change upon selective binding, we used a modified version of this sequence which converted the aptamer into a hairpin structure by adding seven nucleotides at the 3′-end (see ESI,† scheme Fig. E2). Similar designs have been included in numerous optical and electrochemical sensor applications where it was proven that a major structural rearrangement takes place upon binding to the ligand.^11^ Our own previous studies indicated that the average structural rearrangement of such aptamer-hairpin films was in the order of 1.6 nm.^12^ As a negative control for the selective response of the self-assembled membrane, we exchanged the ATP-aptamer by a mutated form (CACCTAGGAGAGTAATGCCGAGGAAGGTTCCAGGTG) which differed in only four nucleotides from the original sequence leading, however, to a practical non-specificity toward ATP.^13^

**Fig. 1:**
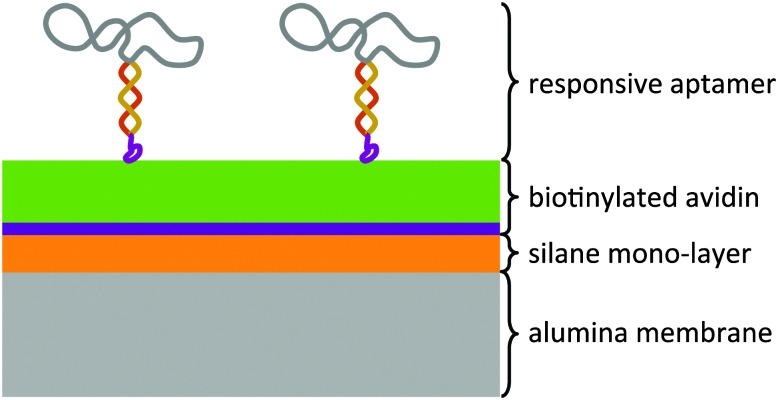
Modular architecture of the small-molecule stimuli-responsive membrane. The thickness of the support structure comprising the silane and avidin layers may be adapted depending on the size of the responsive receptor molecule (aptamer) and the pore size of the base material (AAO membrane).

The stimuli-responsiveness of the membrane was tested by using a permeation cell of 20 mm^2^ effective membrane area. The cell was designed such as to allow facile exchange of the upstream (feed) solution while continuously recirculating a receiving buffer solution over the downstream (permeate) side of the membrane (see ESI,† Fig. E3). We tested the selective responsiveness of the membrane and, hence, modulation of membrane permeability by placing the ATP-aptamer modified membrane in a small diffusion cell with a recirculating permeate buffer solution (for experimental set-up see ESI†). A typical experiment started with buffer solution on both sides of the membrane for monitoring the baseline. At a given time, we added fluorescein sodium salt as a tracer with a molar mass of 37 627 g mol^–1^ to the feed solution yielding a concentration of 3 nM, and subsequently measured its equilibrium concentration in the recirculating permeate on-line using a single-photon counting spectrofluorimeter. The experiment consisted in replicas of three sets of experiments: (1) responsiveness of the membrane to different ATP-target concentrations when modified with the non-specific mutated ATP-aptamer (test for true negative); (2) responsiveness of the ATP-aptamer modified membrane to increasing concentrations of the target ATP (true positive); (3) specificity of the ATP-aptamer modified membrane for ATP *versus* GTP; the chemical structure of GTP is very similar to that of ATP, but the ATP-binding aptamer used in our study is known to practically not bind to GTP, at all.^6^

As can be seen from Fig. 2, the mutated ATP-aptamer modified membrane did not show any significant pore-closing in response to increasing concentrations of the ATP target in the feed solution. In stark contrast, the ATP-aptamer modified membrane not only responded to the presence of ATP in the feed solution, but the response was also ATP-target concentration dependent. The dissociation constant of the ATP-aptamer used in this study has been determined to be around 345 μM for ATP, which is in very good agreement with the actuation of the stimulus-responsive membrane: it shows 50% of the maximum observed pores closing precisely around that concentration, in between 250–500 μM of ATP in solution. The fact that the mutated ATP-aptamer modified membrane did not respond to ATP, at all, corroborates the observation that it is indeed the molecular recognition event between the ATP-aptamer and its target which triggers pore closing: the minor difference in four nucleotides between mutated and ATP-aptamer warrants that both the pore architecture as well as the surface chemistry are practically identical and any possible experimental artefacts such as electrostatic interactions can in this way be comfortably ruled out.

**Fig. 2:**
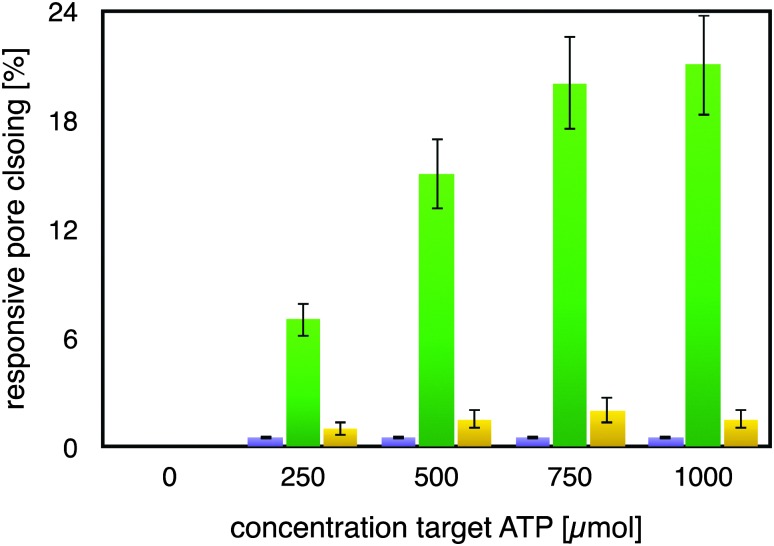
Modulation of membrane permeability, represented by degree of pore closing, through specific recognition of ATP (green) by the ATP-aptamer modified AAO-membrane. Only a minor non-specific responsiveness was observed toward GTP (yellow); the AAO-membrane modified with a mutated ATP-aptamer exhibited a negligible response upon exposure to the ATP-target (violet).

Indeed, this reveals one of the strengths of using DNA-aptamers as gating elements, namely their facile pointwise chemical modification which allows a straightforward, systematic exploration of their actuation. The ATP-modified membrane also responded only to a minor degree and non-specifically to the addition of GTP to the feed solution, confirming its specificity to ATP. As a matter of fact, the membrane responsiveness and specificity were in very good agreement with previous binding studies on the binding of an ATP-aptamer molecular beacon to ATP and GTP, respectively (ESI,† Fig. E4). Reversibility of our small-molecule responsive membrane was of paramount importance for being used in nanodevices; replicas of the experiments were conducted by repeatedly exchanging the feed solution containing either ATP or GTP, respectively, with buffer solution. Error bars in Fig. 2 depict the variation of the experimental results so obtained which amounted to up to 15% from the respective mean pore closing value. Such a variation is not surprising considering that the pore-closing of the membrane is an average responsiveness of an ATP-aptamer film within a not entirely isoporous support structure.

Two aspects were fundamental for the proof-of-concept of this ATP-aptamer gating membrane which is responsive to a molecular rather than a bulk stimulus: first, the target (ATP) is of similar molecular size as the tracer molecule, fluorescein. We therefore could expect that the target would permeate as freely through the membrane pores as the tracer, and in this way reach any available ATP-aptamer recognition sites. Second, ATP with a molar mass of 50 718 g mol^–1^ may still be considered a small molecule; the pore closing observed upon its binding to the ATP-aptamer therefore could be fully attributed to a conformational change of the latter. In the case of targets being significantly larger molecules, pore closing would possibly have to be attributed at least partially to the pore blocking by the bound target itself and in this way not fully prove the concept that conformational changes of functional DNA can bring about changes in permeability across synthetic membrane pores. Fig. 3 clearly indicates that the pore closing of about 20% as compared to the reference state is far from being complete. There is an obvious need to go beyond our proof-of-concept and optimize the system as the dimensions of the conformational change of the DNA-aptamer and the nominal pore size of the membrane support are interdependent parameters. Polymer membranes of a significantly higher isoporosity^14^ than the AAO-membranes used in this study are expected to allow a better control over the design and are currently under study. In an attempt to rationalize the maximum degree of pore closing we could expect from the responsive system described here, we resorted to additional measurements (see ESI†) on the average thickness of the layers employed in assembling the responsive membrane, schematically represented in Fig. 3.

**Fig. 3:**
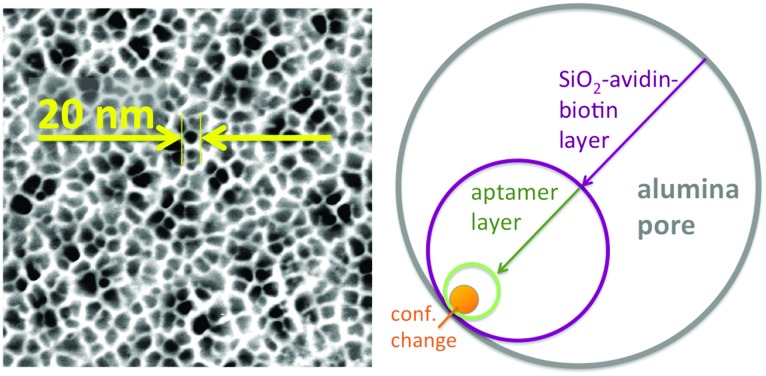
Left: SEM picture of pore-structure of the AAO-membrane with a nominal pore size of 20 nm in its pristine form; right: schematic on the effect of the gradual membrane surface modifications on the effective pore-size.

The average pore diameter of the pristine AAO membrane was determined to be in the range of 20 nm. In previous work, it was shown that under the experimental conditions described here the deposition of avidin yields a layer of around 5 nm thickness.^12^ Subsequent deposition of a biotinylated aptamer-hairpin resulted in a further average film thickness increase of around 3.5 nm, such that the resulting decreased pore diameter can be assumed to be at the order of about 3 nm corresponding to a pore area of about 7 nm^2^. This compares favourably with the average pore diameter of about 2.3 nm of mesoporous silica particles that have proven to be efficient controlled release platforms when modified with a similar ATP-aptamer.^6,10^ The average thickness change of an ATP-aptamer hairpin film upon exposure to its target was previously determined to be in the order of 1.6 nm. Since ATP-aptamers will not be tightly packed as a dense layer owing to their negative charge but rather resemble brushes at the mouth of the pore, this thickness change cannot be directly translated into blocking of the pore as would be expected upon the swelling of a dense polymer. As an approximation, we therefore associated this thickness change with an average actuation diameter resulting in an actuation area that represents the additional effective blocking of the pore upon target recognition (Fig. 3). Based on a diameter of 1.6 nm, this area would amount to 2 nm^2^ corresponding to about 30% of the overall reduced free pore area of 7 nm^2^ without target recognition, comparing very favourably with the measured maximum pore closing values of around 20% upon exposure of the stimulus-responsive membrane to the target, ATP (Fig. 2). This merely size-related argumentation does not consider possible charge effects which might further affect the permeation of the negatively charged fluorescein across the aptamer-modified nanopore. The negative charge of the DNA-aptamer might be exposed to a different extent depending on the aptamer conformation, which can further enhance the modulation effect. This aspect is currently subject of further studies using uncharged reporter molecules.

This proof-of-concept study demonstrated the feasibility of using DNA-aptamers as specific, reversible, and target-concentration dependent actuators in membranes whose permeability can be modulated through a molecular recognition event rather than a bulk stimulus. Such membranes find a great application potential in biomedical and bioanalytical flow devices.

T. Schäfer would like to gratefully acknowledge ERC Starting Grant 209842-MATRIX, and thank P. Echenique for being hosted in the Donostia International Physics Centre (DIPC), San Sebastián, Spain. T. Schäfer thanks A. Chuvilin and E. Nikulina, CIC Nanogune Donostia-San Sebastián, for the SEM picture of the AAO-membrane.
