# Supplementary material for: DNA-aptamer gating membranes
Source: Chem Commun (Camb). 2015 Jan 30;51(25):5471–4. doi: 10.1039/c4cc09660f (PMC4840836; doi:10.1039/c4cc09660f)
Supplement: Supplementary file 1 [file CC-051-C4CC09660F-s001.pdf]

Electronic Supplementary Material (ESI) for ChemComm.
This journal is © The Royal Society of Chemistry 2015

Electronic Supplementary Information

DNA-aptamer gating membranes

Thomas Schäfer*[a,b] and Veli Cengiz Özalp[c]

[a] Polymat, University of the Basque Country, Av. Tolosa 72, 20018 Donostia-San Sebastián, Spain
[b] Ikerbasque, Basque Foundation for Science, Spain
[c] School of Medicine, Istanbul Kemerburgaz University, 34217 Istanbul, Turkey

(1) Surface Modification of the Anodisc Alumina Oxide (AAO) Membranes

The aptamer-modified AAO membranes were prepared by a procedure of sequential depositing the monolayers of propylamylethoxysilane, covalent attachment of BS4, and finally attachment of biotinylated aptamer sequences through an avidin sandwich. All reagents were obtained from Sigma-Aldrich and used as received, unless stated otherwise. The details of layer-by-layer procedure involved the following steps:

i) Silanization of the alumina surface:

The 20 nm pore-size AAO (Whatman) membranes were functionalized with amino groups through an ethoxysilane monolayer deposition. The membrane discs were cleaned in 37 % peroxide followed by UV-ozone treatment. The clean membranes were incubated for 30 min. at room temperature in a 4 % solution of 3-aminopropyltriethoxysilane in a 5 % water in ethanol mixture, acidified to yield pH=4.5 with acetic acid. The membrane was the washed several times with ethanol and dried under vacuum at room temperature.

ii) Surface functionalization with biotin:

Biotinylated *N*-hydroxysuccinimide ester of a polyethylene glycol spacer arm (EZ-Link NHS-PEG4-Biotin, Piercenet) was attached to amino groups on the surface of the silanized Anodiscs through incubation for 30 minutes at room temperature and subsequent washing with PBS-buffer.

iii) Immobilization of the biotinylated ATP-binding aptamer:

The biotinylated anodisc surface was incubated for 4 hours in avidin solution and subsequently another 4 hours in biotinylated aptamer solution in PBS-buffer. The aptamer-functionalized membranes were air-dried and kept at 4 °C until used for the experiments.

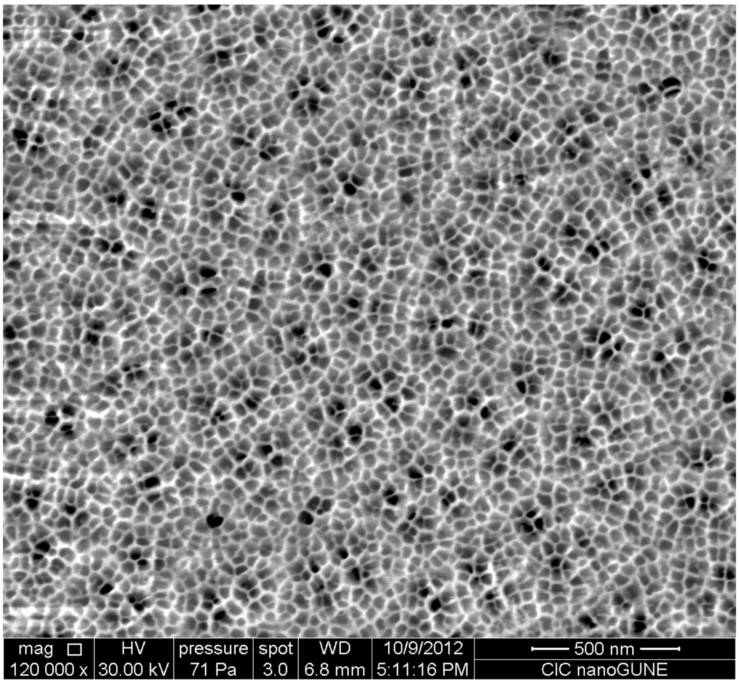

Fig. E1 SEM-image of the AAO-membrane illustrating the well-known high porosity of such membranes; nominal pore diameter: 20 nm. The photograph also reveals that apparently many pores are discontinuous as might be deduced from the light grey shades.

(2) Schematic of the modulation of the permeability across a nanopore through conformational change of ATP-aptamer:

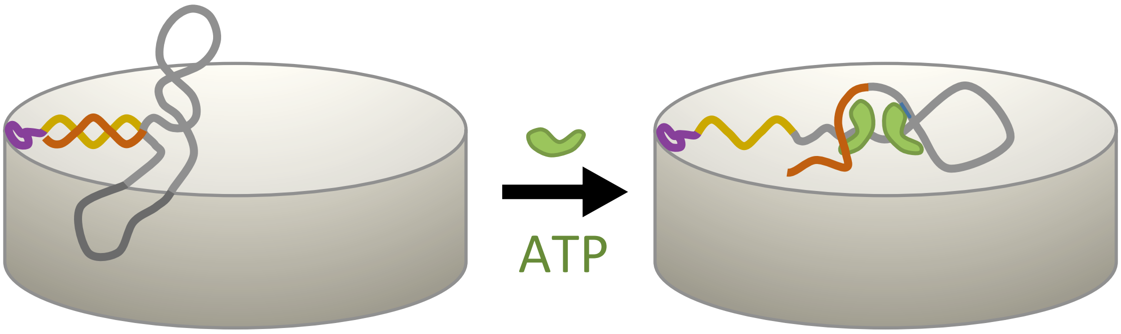

Fig. E2 Schematic of the conformational change which the ATP-binding aptamer undergoes upon molecular recognition of ATP in the membrane pore. The aptamer consisted of the specific base sequence (grey), added nucleotides for creating a hair-pin structure[13] (yellow) and a biotin-linker (violet). Specific binding to ATP results in an opening of the hairpin and a significant conformational change of the aptamer within the membrane pore. The conformational change has previously been quantified to be at the order of about 1.6 nm.[12]

(3) AAO-membrane surface modification: QCM-D measurements

Immobilisation of avidin/ATP-aptamer on aluminum oxide

The efficiency of the AAO-membrane surface modification through avidin and subsequent ATP-aptamer immobilization was confirmed by a quartz crystal microbalance with dissipation monitoring. All experiments were carried out in PBS-buffer (0.01 M phosphate buffered saline; NaCl: 138 mM; KCl: 2.7 mM; $MgCl_2$: 5 mM; pH 7.4) at a constant temperature of 23±0.1°C. Aluminum oxide coated quartz sensors were purchased from Q-SENSE (Biolin Scientific/Q-Sense AB, Västra Frölunda, Sweden) and the QCM-D measurements performed with a Q-Sense E1 system (Biolin Scientific).4

The average coverage of avidin followed by the ATP-aptamer is given below. The molar coverage is a rough approximation assuming a density of 1350 kg/dm^3 for both avidin and the ATP-aptamer[12] as well as a molar weight of 67 kDa and 11,26 kDa for avidin and the ATP-aptamer, respectively:

Table1: Frequency change (ΔF), corresponding mass (Δm), thickness (Δd) and molar (Δn) change per area during the adsorption of avidin and ATP-aptamer, respectively, on a aluminum oxide quartz sensor:

	ΔF [Hz]	Δm [ng/cm^2]	Δd [nm]	approx. Δn [nmol/cm^2]
avidin	38±3	673±53	5.0	0.01
ATP-aptamer	28±4	496±71	3.6	0.04

The data in the last column indicate that the avidin/ATP-aptamer interaction was indeed in the range of the maximum number of possible interactions (four). It can be expected that within the porous structure of the AAO membranes the effective binding efficiency of the aptamer to the avidin sub-layer might be lower than during the QCM-D analysis which uses controlled flow conditions over a non-porous surface.

(4) Permeability measurements

A permeation flow cell of an efficient membrane diameter of 5 mm was employed. The membrane was sandwiched between the upstream (feed) chamber (green chamber on top) and the downstream (permeate) chamber which provided a continuous flow of buffer solution. The permeate stream was recirculated through a single-photon counting spectrofluorimeter ("SPC SF", FLS920, Edinburgh Instruments) for the on-line analysis of fluorescence (Exc. 480 nm, Em. 520 nm, 450-W xenon arc lamp).

At the beginning of the experiment, the feed chamber was filled with buffer solution for stabilizing the system and the fluorescence baseline monitored (background). Subsequently, the feed solution was substituted with a 3 nM fluorescein sodium salt solution and the baseline again monitored (maximum aperture of the pore). Once a stable baseline was obtained, the feed solution was exchanged such as to yield the respective ATP and GTP concentrations. All solutions were strictly based on the very same fluorescein stock solution such as to avoid experimental artifacts through varying fluorescein concentrations in the feed.

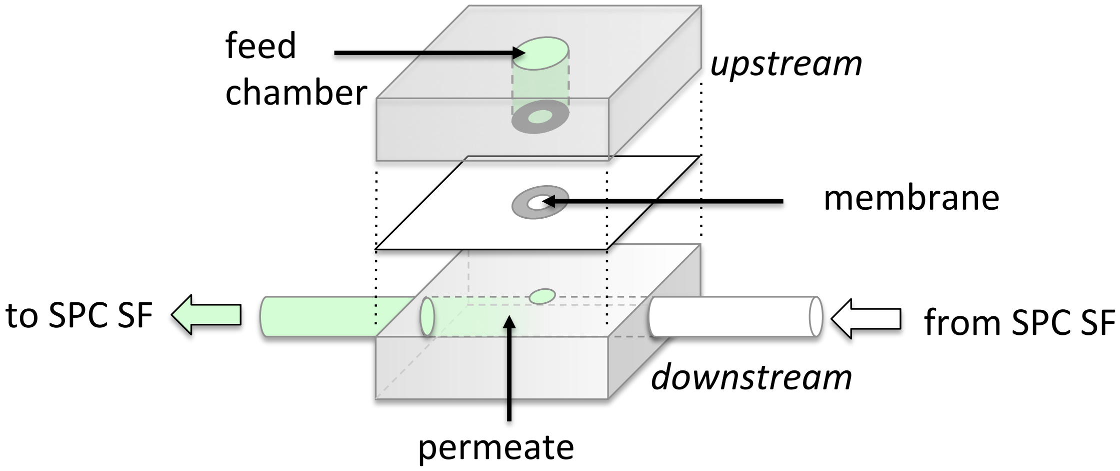

Fig. E3 Schematic of the miniature permeation flow cell for measuring the stimuli-responsiveness of the ATP-aptamer modified AAO-membrane. Prior to any experiment, the system is stabilized by placing buffer solution into the feed and permeate compartment. Upon adding fluorescein to the feed buffer solution, the former diffuses according to a concentration gradient across the membrane into the permeate buffer solution which is recirculated through the cuvette of the spectrofluorimeter for on-line analysis of the fluorescence. The volume of the recirculation circuit was strictly minimized.

(5) Correlation between binding studies of the ATP-aptamer hairpin in solution and the pore-closing observed in the ATP-aptamer modified responsive membrane:

The K_D of the ATP-aptamer hairpin employed in this study is around 345 μM in solution and presence of its target, adenosine-5'-triphosphate (ATP), while it is virtually non-responsive to guanosine-5'-triphosphate (ATP).[12] This specificity reflects in the responsiveness of the ATP-aptamer modified AAO-membrane: Fig. E4 represents the pore closing observed in this study at defined ATP-concentrations with the normalized fluorescence observed previously with an ATP-molecular beacon at identical concentrations. As can be seen, the maximum pore closing is observed when the normalized fluorescence is maximum, i.e., when the ATP-aptamer beacon is opened most going along with a major conformational change. In contrast, at low fluorescence data (=molecular beacon closed = no target recognition = no conformational change) also the pores remain almost fully open. Hence, pore closing is strongly correlated with ATP-binding (represented by increasing normalized fluorescence).

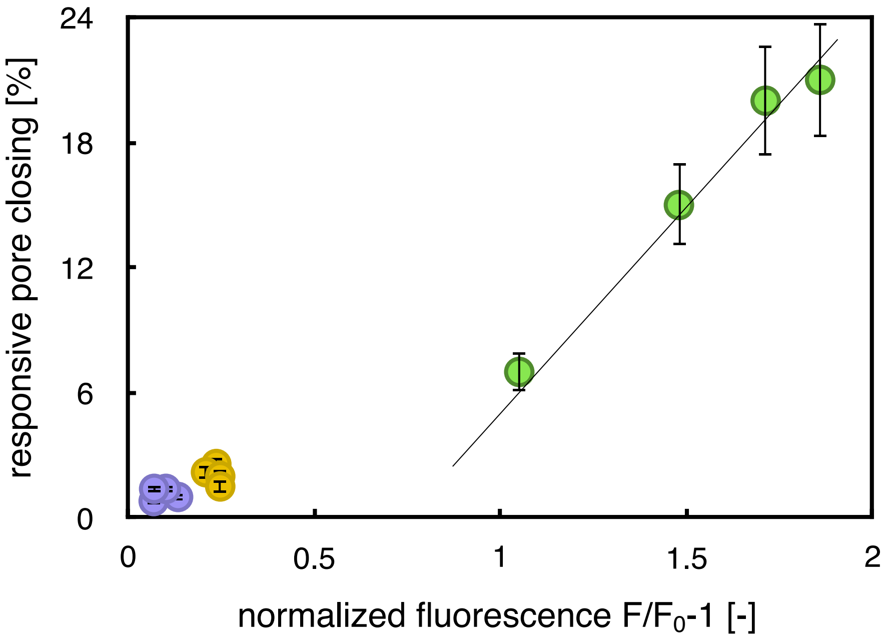

Fig. E4 Correlation of the responsiveness of the ATP-aptamer modified AAO-membrane (represented as pore closing) and the ATP-binding as observed with an ATP-aptamer molecular beacon. The origin of the graph represents the reference situation of maximum pore aperture in absence of any target (ATP); violet: mutated ATP-aptamer; yellow: ATP-aptamer with GTP as non-specific target; green: ATP-aptamer with specific target ATP.
